# Modified nanofat grafting: Stromal vascular fraction simple and efficient mechanical isolation technique and perspectives in clinical recellularization applications

**DOI:** 10.3389/fbioe.2022.895735

**Published:** 2022-09-13

**Authors:** Paul Girard, Joelle Dulong, Jerome Duisit, Camille Mocquard, Simon Le Gallou, Benoit Chaput, Elise Lupon, Eric Watier, Audrey Varin, Karin Tarte, Nicolas Bertheuil

**Affiliations:** ^1^ Department of Plastic, Reconstructive and Aesthetic Surgery, CHU Rennes, University of Rennes I, Rennes, France; ^2^ INSERM U1236, University of Rennes I, Rennes, France; ^3^ SITI Laboratory, CHU Rennes, Rennes, France; ^4^ Department of Plastic, Reconstructive and Aesthetic Surgery, Rangueil Hospital, CHU Toulouse, Toulouse, France; ^5^ INSERM U1031 STROMALab, Toulouse, France

**Keywords:** mechanical isolation, adipose tissue, SVF, recellularization, clinical applicability, adipose derived stem cells, nanofat, immunomodulatory properties

## Abstract

**Background:** Nanofat grafting (NG) is a simple and cost-effective method of lipoaspirates with inter-syringe passages, to produce stromal vascular fraction (SVF) and isolate adipose-derived stem cells (ASCs). This represents a tremendous interest in the future clinical needs of tissue engineering. In this study, we optimized the NG technique to increase the yield of ASC extractions.

**Methods:** We analyzed three groups of SVF obtained by 20, 30, and 40 inter-syringe passages. The control group was an SVF obtained by enzymatic digestion with Celase. We studied their cell composition by flow cytometry, observed their architecture by confocal microscopy, and observed immunomodulatory properties of the ASCs from each of the SVFs by measuring inflammatory markers of macrophages obtained by an ASC monocyte co-culture.

**Results:** We have established the first cell mapping of the stromal vascular fraction of adipose tissue. The results showed that SVF obtained by 20 inter-syringe passages contains more statistically significant total cells, more cells expressing the ASC phenotype, more endothelial cells, and produces more CFU-F than the SVF obtained by 30 and 40 passages and by enzymatic digestion. Confocal microscopy showed the presence of residual adipocytes in SVF obtained by inter-syringe passages but not by enzymatic digestion. The functional study indicates an orientation toward a more anti-inflammatory profile and homogenization of their immunomodulatory properties.

**Conclusion:** This study places mechanically dissociated SVF in the center of approaches to easily extract ASCs and a wide variety and number of other progenitor cells, immediately available in a clinical setting to provide both the amount and quality of cells required for decellularized tissues.

## Introduction

Among current tissue engineering strategies to produce high-quality scaffolds, the so-called decellularization technique is the only technique yet to fulfill this goal (Badylak, 2002). Natural tissue-derived extracellular matrix (ECM), thus, brings the most complex 3D architecture, extensive components, and associated growth factors, with applications to a wide variety of simple to complex tissues, and organs (Crapo et al., 2011). The recellularization aspect, however, remains a huge challenge in order to reach clinical relevance, due to several limiting factors: the type and number of cells to be cultured, the cost of personalized medicine, and associated regulatory issues. As stated by Badylak (2016), the most realistic and applicable approach would be to use the human body's regenerative potential. Identification of adipose stromal cells (ASCs) (Zuk et al., 2002) within the adipose tissue (AT) made it valuable for clinical regenerative purposes (Wang et al., 2013). However, isolation and expansion of ASCs remain a long and expensive process, with the need for a laboratory step before its use back in a patient. This is the reason why stromal vascular fraction (SVF), a direct and easy source of ASCs and various cells types—like endothelial progenitor cells, smooth muscle cells, pericytes, M2 monocytes/macrophages, and regulatory T cells—appears to be a particularly promising aspect in the field. With already several examples of recellularization strategies based on SVF (Scarritt et al., 2015; Duisit et al., 2018; Tan et al., 2019; Gentile et al., 2020), SVF obtention and clinical handling have already been extensively studied in the field of plastic surgery, in particular through the nanofat grafting (NG) technique (Tonnard et al., 2013). The reference method to isolate SVF is enzymatic digestion (Aronowitz and Ellenhorn, 2013; Raposio et al., 2014). Some commercial devices are now available to produce SVF inside an operating room, but the process remains time-consuming and expensive (Aronowitz and Ellenhorn, 2013). Mechanical isolation of SVF with current devices represents a seducing alternative to the operating room, faster and cheaper than enzymatic digestion (Tonnard et al., 2013; Raposio et al., 2016; Mashiko et al., 2017). Chaput et al. (2016) showed that ASCs produced by mechanical isolation matched Dominici’s criteria (Dominici et al., 2006; Bertheuil et al., 2015, 2019) and were able to inhibit T-cell proliferation (Chaput et al., 2016). Since this study, it is now clear that the most interesting technique in a clinical setting is the NG technique (Tonnard et al., 2013), performing an AT emulsification through 30 intersyringe passages. It is a simple, reproducible, and cheaper procedure, but the scientific evidence that ASC yield was improved had not been given yet. Furthermore, Tonnard et al. (2013) initially stated that SVF resulting from the NG technique contained no adipocytes later; Lo Furno et al. (2017) showed that nanofat contains fewer adipocytes instead of none. Due to the very high potential of SVF as a cell reservoir for regenerative medicine and recellularization purposes, we focused on extending the scientific roundness of its bedside extracting techniques. The objectives of this study are as follows: 1) to optimize the NG technique and determine, with flow cytometry, the closest number of intersyringe passages to maximize SVF cellular composition and functionality; 2) to compare SVF architectural changes between mechanical and enzymatic digestions, as well as the presence of adipocytes, by confocal microscopy; 3) to study, by co-culture and flow cytometry, the immunomodulatory properties of different SVF-isolated ASCs toward macrophages, key players in tissue regeneration, especially the M2 anti-inflammatory type.

## Material and methods

Between March and July 2018, we included nine female patients undergoing an abdominoplasty procedure in the Department of Plastic Surgery of the University Hospital of Rennes, France. This study was approved by the Institutional review board and performed in accordance with the principles of the Declaration of Helsinki (1964, and the French bioethics laws of July 7th, 2011, after written informed consent from all patients. The mean age of patients was 42.7 years ( ±12.9 SD), and the mean BMI was 26.35 kg/m^2^ ( ±3.2 SD). AT was manually harvested using 4-mm-diameter cannulas with a 400-mmHg vacuum, before starting the abdominoplasty procedure. We aimed at comparing the group of mechanically extracted SVF to a control group of enzymatically isolated SVF.

### Management of AT

For each group, a 10 g sample of AT was processed into SVF, according to its designated technique. The final product was washed with a phosphate-buffered saline (PBS, 14,040, Life Technologies, California, United States) solution and centrifuged at 680 g for 5 min. Cell pellets were collected to count the number of cells in each sample. We normalized the count of cells of each SVF per gram of initial AT.

### Mechanical isolation

The AT sample was transferred into a 10-ml syringe with a Luer lock nozzle connected to an identical syringe, with a 3-way connector (Connecta Luer Lock, ref 394,601, Becton Dickinson, New Jersey, United States ). AT was emulsified by 20 (P20), 30 (P30), or 40 (P40) intersyringe passages, but with no filtration at 500 µm, contrary to Tonnard’s original technique (Tonnard et al., 2013). Mechanically isolated SVF suspensions were stored in 50-ml tubes until further processing.

### Enzymatic digestion

AT was digested with 1 ml of PBS and 18 µl of Celase^®^ (final concentration of 0.09 mg/ml) per gram of tissue for 25 min at 37°C, as recommended. The enzyme was neutralized with PBS, and the digested tissue was filtered through a 100-μm Steriflip^®^ unit (SCNY00100 Merck Millipore, Massachusetts, United States), and it was then centrifuged at 680 g at ambient temperature for 10 min.

### Conditioning of samples before analysis

In order to precisely analyze cell composition and avoid a bias between the two isolation processes, an additional extensive enzymatic digestion process was applied to the two groups: the objective was to obtain a fully digested cellular suspension, to be analyzed by flow cytometry after filtration. Thus, each sample from mechanically isolated SVF and enzymatically isolated SVF was digested for 45 min at 37°C on a shaker using a solution with 10 ml α-MEM (32,561, Life Technologies), 100 UI/mL, and 100 μg/ml penicillin/streptomycin (15140122, Life Technologies), 200 UI/mL, 100 UI/mL, and 100 μg/ml penicillin/streptomycin (15140122, Life Technologies), 200 UI/mL of type IV CLS-4 collagenase (rLS00189, Worthington), 1.6 UI/mL dispase (LS02104, Worthington), 10 UI/ml DNase (Pulmozyme, ref 10139247, Roche, Basel, Switzerland), and 5 mM MgCl, (magnesium chloride, ref AM95306, Ambion, Thermo Fisher Scientific, Massachusetts, United States). At the end of the digestion process, all samples were washed with PBS and filtered at 100 µm, as before, and the pellet was transferred in a PBS and 10 U/ml of DNase solution, with a volume of 1 ml per gram of freshly harvested tissue.

### Immunophenotypic analysis

Each SVF sample was then centrifuged, and cell pellets were re-suspended in 300 μl of 1% PBS–albumin (ref BE17-512 F Lonza, Basel, Switzerland). Samples were incubated for 10 min at 4°C with FVS-780 (ref 565,388, BD Biosciences, United States) to study viability and then incubated with brilliant staining buffer (ref 563794, BD) in order to prevent any interference between Brilliant Violet and Brilliant Ultra Violet fluorochromes. Cells were incubated at 4°C for 25 min using the following antibodies: anti-CD146-BUV395 (ref 564326), anti-CD3^−^ BUV496 (ref 564810), anti-CD45-BUV805 (ref 564916), anti-CD335-BV711 (ref 563043 BD), anti-CD11b-PE (ref PN IM25181U), anti-CD235a-FITC (ref PN IM2212U), anti-CD19-PC7 (ref IM328 Beckmann Coulter, CA, United States ) anti-CD14-PE (ref R0864), anti-CD16- PE (ref R7012, Dako Agilent Technologies, California, United States ), anti-CD15-PB (ref 323022, BioLegend CA, United States ), anti-CD31=PerCP eF710 (ref 46-0319-42 eBioscience Thermo Fisher Scientific MA, United States ), and anti-CD34-APC (ref 130-090-954, Miltenyi Biotec, Germany). After labeling, we performed erythrocyte lysis (ref S2364, Dako), and after a final wash, cell pellets were re-suspended in 240 µl of 1% PBS–albumin with 10 µl of DNAse and 50 µl of beads for counting (ref 424902, Beads Comp, BioLegend). Acquisitions were performed on a multicolor flow cytometer (BD, Fortessa X20, BD Biosciences).

### Clonogenicity

The presence of colony forming unit fibroblasts (CFU-F) was assessed in SVF. Cells were seeded at 8 cells/cm^2^ in 25 cm^2^ plates with 5 ml of culture medium made of αMEM, fetal calf serum 10% (ref FB-1101/500 Bioserra), and penicillin-streptomycin (20 UI/mL final, ref 15140-122 Gibco, Thermo Fisher Scientific). The culture medium was initially replaced after 24 h and then twice a week. Staining took place on day 14 with a MCDH kit (ref 313590–313570 – 313560–313600, RAL Diagnostics, France). Colonies of more than 50 cells were considered CFU-F. The count of ASCs was defined as the number of CFU-F divided by the number of cells initially seeded.

### Confocal microscopy

The objective was to detect residual intact mature adipocytes in mechanically isolated SVF. Adipocytes were stained with BODIPY 558/568 1/50 dilution (Invitrogen, Thermo Fisher Scientific), endothelial cells with Alexa Fluor 488-Isolectin 1/50 dilution (I21411 Invitrogen, Thermo Fisher Scientific), and cellular nucleus with DAPI 1/50 dilution (D9542-1 MG Sigma Life Science, Missouri, United States). We used a “whole-mount staining” protocol based on a technique described by Mashiko et al. (2017). Preparations were fixed with 4% paraformaldehyde with shaking for 10 min then rinsed three times with PBS. Then, preparations were incubated for 1 h with a saturation solution of PBS, saponin 0.01% final (S7900, Quillaja Bark Sigma life science), 2% bovine serum albumin (A3294 Sigma Life Science), and 4% donkey serum (CO6SB, Bio-Rad, California, United States). Staining was performed on approximately 500 μl of tissue for 30 min at room temperature while shaking. Tissue was placed on a microscopic slide using the “thick smear” technique. After a 12-h drying process, slides were observed by confocal fluorescent SP8 microscopy (Leica, Germany).

### Immunomodulatory assay

The objective was to assess the *in vitro* functional ability of SVF-derived ASCs to polarize macrophages. The monocytes used were, therefore, stored in liquid nitrogen, obtained by elutriation of leuko-platelet concentrate from discarded banked human blood. At the end of passage 0, ASCs were co-cultured with monocytes at a ratio of 4:1 in RPMI (ref 61870-010, Life Technologies), fetal calf serum (ref FB-1101/500, Bioserra), penicillin-streptomycin 20 UI/mL final (ref 15140-122, Gibco), and M-CSF 100 ng/ml final (ref 216-MC-005 R&D Systems, Minnesota, United States). After 5 days, cocultures were stopped, and cells were stained with anti-CD45^−^ BUV395 (ref 56379 BD), anti-CD73-BV786 (ref 7423635 BD), anti-CD206-APC (ref 550889 BD), anti-CD163-PE (ref 556018 BD), and anti-HLA-DR-BV605 (ref 562844 BD). Cells were analyzed in a multicolor flow cytometer. The control group was defined for M1/M2 differentiation: in order to create a reference scale of macrophage polarization, we performed an M1 and M2 differentiation *in vitro,* on the same batch of monocytes we used for the co-culture experiments. To promote differentiation, we added cytokines to the culture medium: GM-CSF (granulocyte macrophage colony-stimulating factor; 200 ng/ml) for M1 wells and M-CSF (200 ng/ml) for M2 wells. The plate was placed at 37°C for 5 days. On day 5, the culture medium of M1 macrophages received IFNg (40 ng/ml) and LPS (200 ng/ml), and the culture medium of M2 macrophages received IL4 (40 ng/ml), IL10 (40 ng/ml) and IL13 (40ng/ml). The plates were placed at 37°C for additional 3 days. The analysis took place on day 8 using the same panel we used in the co-culture. The mean fluorescence intensity of CD206, CD163, and HLA-DR of Fluorescence Minus One (FMO) tubes and TEST tubes was calculated on a monoparametric diagram.

### Statistics

Statistical analysis was performed by GraphPad Prism 7.04 (GraphPad Software, GSL Biotech LLC, CA, United States ). We used a one-factor variance analysis test (one-way ANOVA) for comparison of conditions with one parameter and a Wilcoxon *t*-test for comparison of two versus two conditions. Results are shown with their standard deviations (SD). Graphic representation is shown with scatter plots. Dispersion is represented by the mean standard deviation (meanSD).

## Results

### Flow cytometry

**FIGURE 1:**
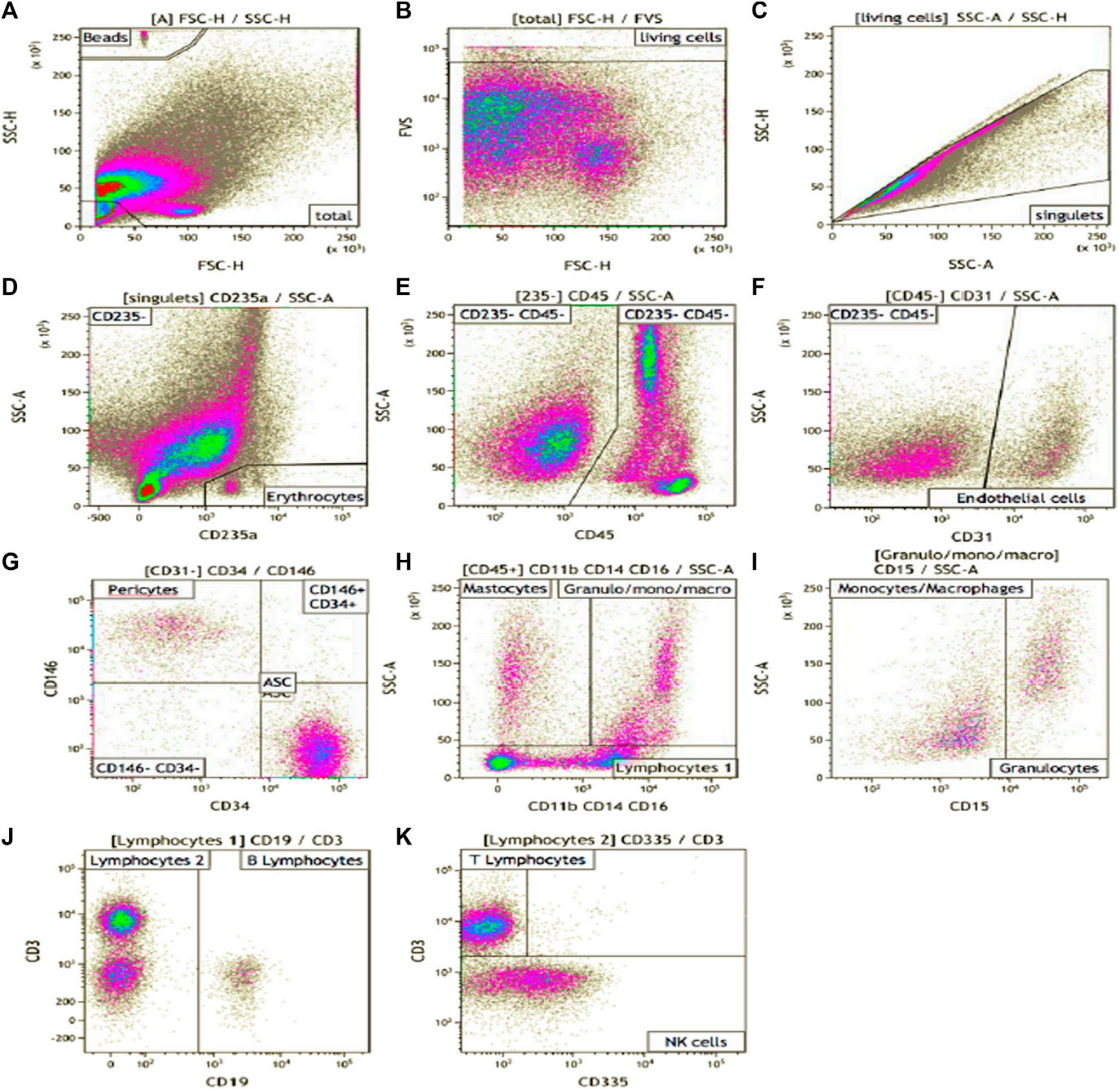
SVF phenotype gating strategy. Manual analysis on Kalooza software. **(A)** showed the gating of beads count. FVS-780 was the viability marker **(B)**. **(C)** eliminate the doublets. CD235a stained erythrocytes **(D)**. CD45 was expressed by hematopoietic cells **(E)**. CD31 was expressed by endothelial cells **(F)**. CD146 stained pericytes (on CD45^−^ CD31^−^cells) **(G)**. CD34 stained AUCs after an adapted gating strategy **(G)**. CD11b, CD14, and CD16 **(H)** were pooled in a cocktail of antibodies on the same filter and stained myeloid cells. CD15 stained granulocytes **(I)**. CD19 stained B cells **(J)**, CD3 stained the T cells **(K)**. CD335 stained NK cells **(K)**.

**FIGURE 2:**
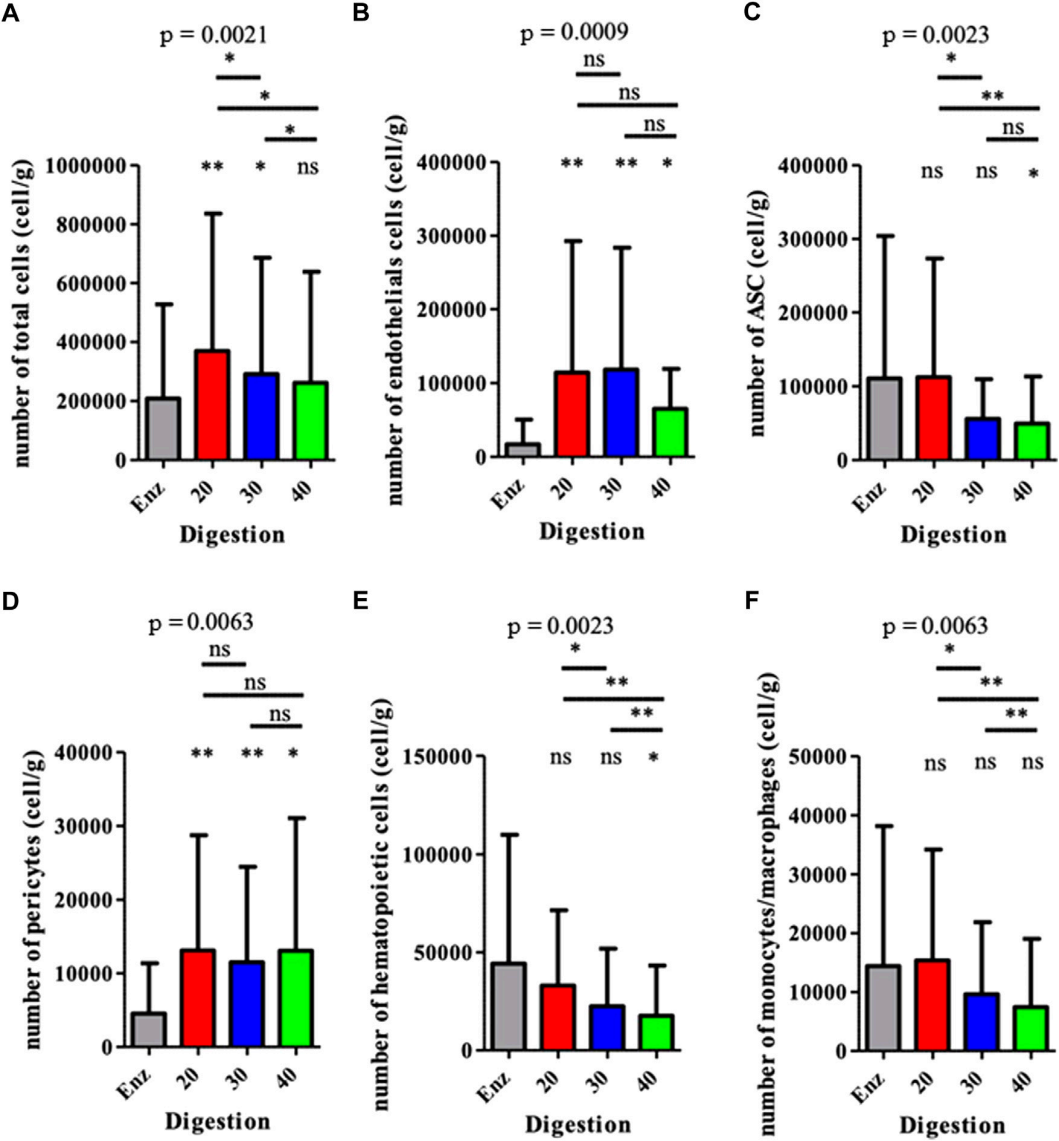
Analysis of the cell populations from each SVF obtained for various methods. The results are expressed as the number of cells per gram of the initial fresh tissue. *p < 0.05, **p < 0.001, and ***p < 0.0001; ns: not significant. SVF: stromal vascular fraction.

**FIGURE 3:**
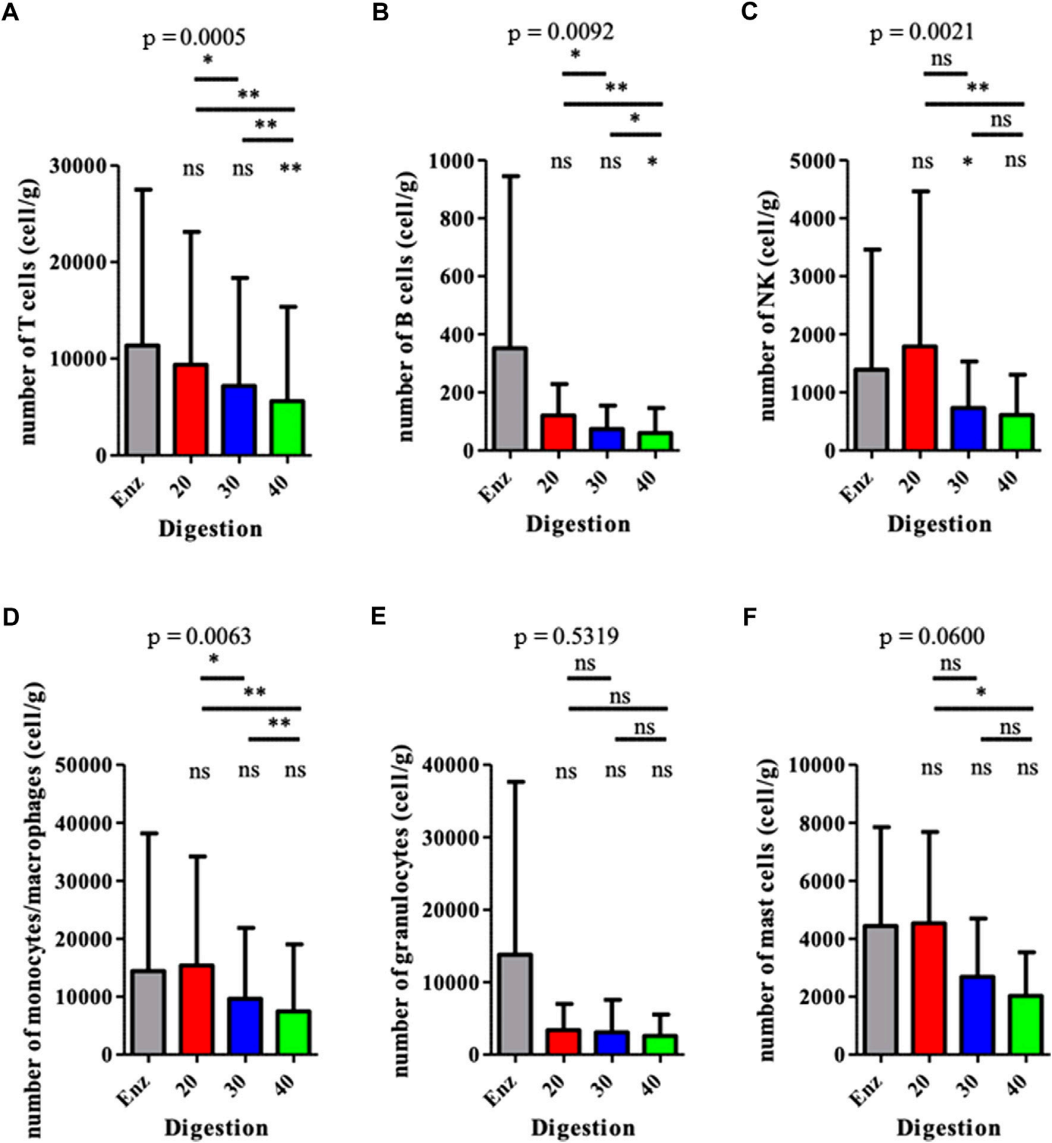
Analysis of the hematopoietic (CD45pos) populations from the SVFs. Results are expressed as a percentage of the total number of cells, for each population. *p < 0.05, **p < 0.001, and ***p < 0.0001; ns: not significant. SVF: stromal vascular fraction.

**FIGURE 4:**
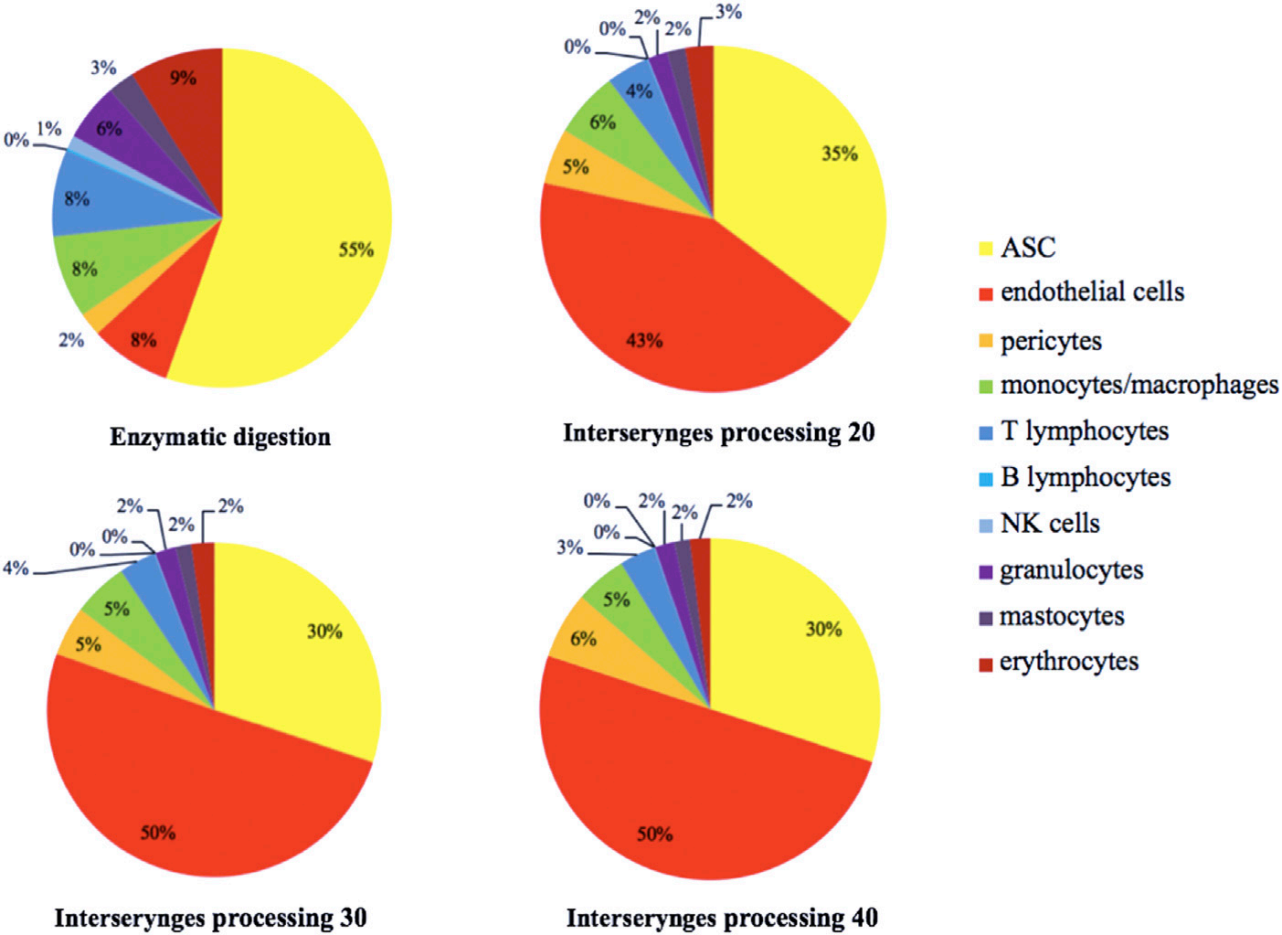
Estimated distribution of the different SVF cell populations, distributed according to the color code indicated. The percentage represents the proportion of each cellular sub-population. SVF: stromal vascular fraction.

The exhaustive cartography of adipose-derived SVF, of mechanically isolated SVF compared to enzymatically isolated SVF samples (Figure 1 **)**, showed important differences between the two groups. However, mechanical M20, M30, and M40 groups presented the same distribution for each cell population, regardless of the number of intersyringe passages. Mechanically isolated SVF with P20 and P30 passages contained a higher number of cells than the enzymatically isolated SVF control group: 369 557 cpg (cells per gram of 167 initial tissue) ± 466 220 (*p* = 0.0078), 291 272 cpg ± 394 588 *p* = 0.0117), and 208 575 cpg ±318 774, respectively (Figure 2A). Mechanically isolated SVF contained more endothelial cells and pericytes than enzymatically isolated SVF (Figures 2B,D). Mechanically isolated SVF P20 had the highest number of cells expressing an ASC phenotype (CD45^−^ CD31^−^ CD34^+^) **(** Figure 2C). There was no difference between the two groups regarding the number of hematopoietic cells, but within the mechanically isolated SVF group, more intersyringe passages decreased their amount **(** Figure 2E **)**; the same observation was made for the number of monocytes/macrophages (Figure 2F **)**. There was no difference between the two groups regarding the number of hematopoietic subpopulation cells (Figure 3 **)**. An increase in intersyringe passages reduced the number of small cells (LT, LB, and NK) (Figures 3A–C) but had no effect on the bigger granulocytes and mastocytes (Figures 3D–F). The proportion of endothelial cells in enzymatically isolated SVF (8.02% ± 6.11) was significantly lower than that in mechanically isolated SVF and following passages: P20 with 43.77% ( ± 16.49, *p* = 0.0039), P30 with 50.09% ( ± 16.42, *p* = 0.0039), and P40 with 51.81% ( ± 20.33, *p* = 0.0039), relying to the proportional increase of other cell populations. Mechanically isolated SVF subgroups had similar repartition of their cell populations (Figure 4).

### Clonogenicity

**FIGURE 5:**
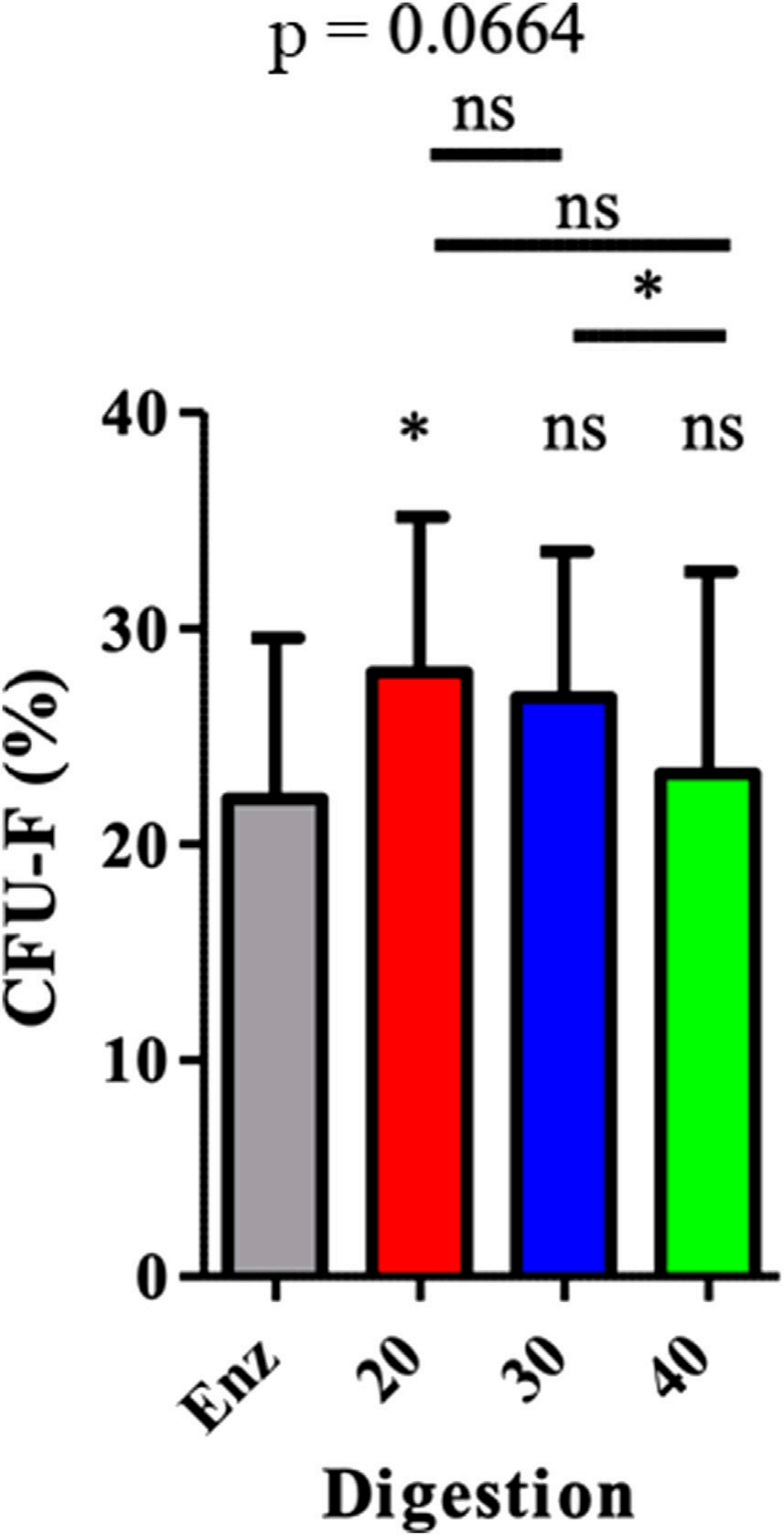
ASC (CFU-F) representation in SVF. The results are expressed as a percentage of CFU-F. It was obtained by the number of CFU-F counted in relation to the number of cells sown. *p < 0.05, **p < 0.001, and ***p < 0.0001; ns = not significant. ASCs: adipose-derived mesenchymal stem cells; CFU-F: colony-forming unit–fibroblast; SVF: stromal vascular fraction.

Mechanically isolated SVF P20 had a significantly higher rate of ASCs than enzymatically isolated SVF of 27.97% (± 7.18) compared to 22.11% (± 7.44) and *p* = 0.0243. (Figure 5).

### Confocal microscopy

**FIGURE 6:**
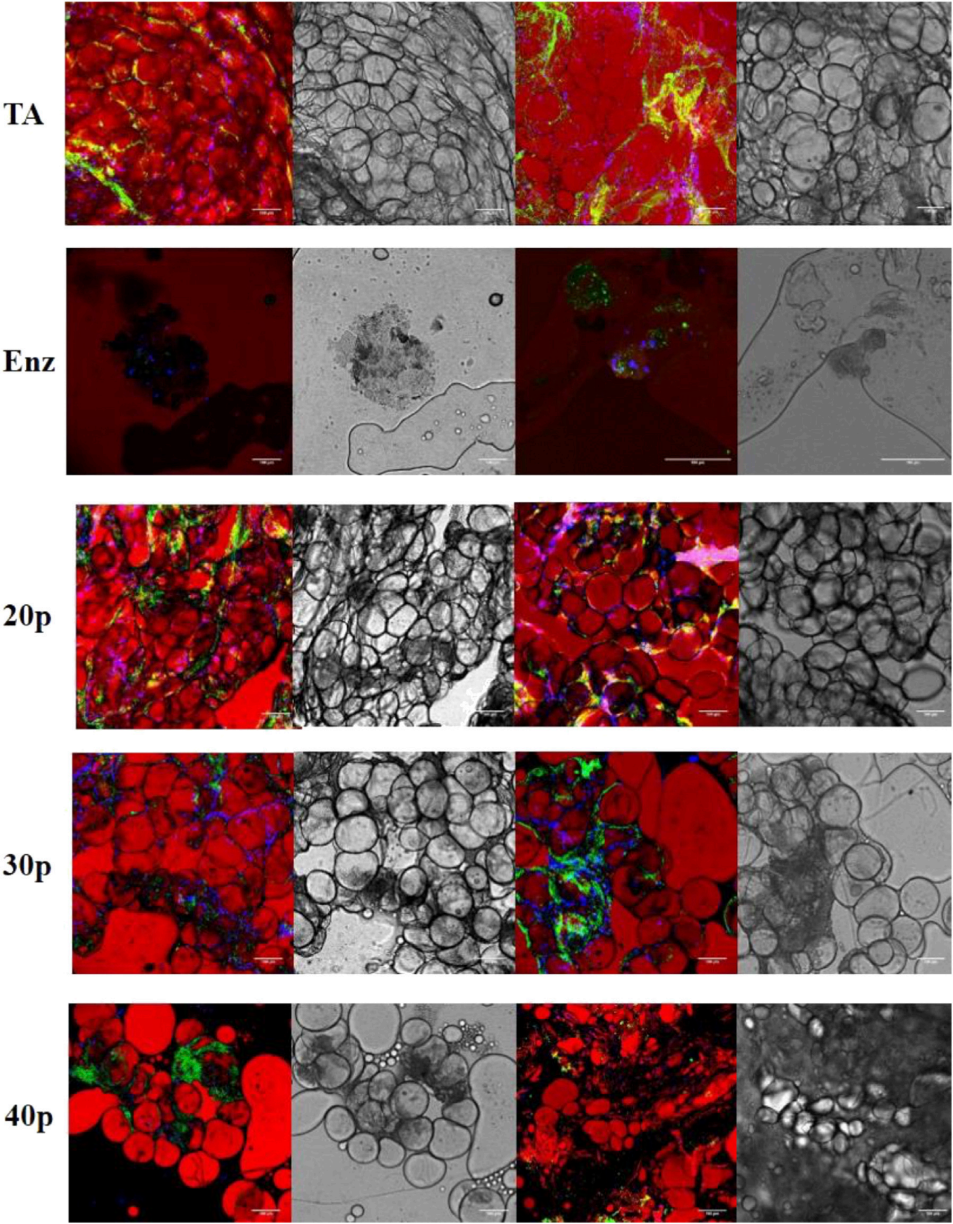
Immunofluorescence by confocal microscopy of the different SVFs. In red, adipocytes are stained by BODIPY; in green, endothelial cells are stained by isolectin coupled with Alexa Fluor 488, and in blue, nucleated cells are stained by DAPI. Shades of gray: transmission electron microscopy imaging of the same section. * False positive, lipid vacuole. SVF: stromal vascular fraction.

In the enzymatically isolated SVF group, confocal microscopic observation with BODIPY staining demonstrated the absence of any intact mature adipocytes. However, in mechanically isolated SVFs, all types of adipocytes (Figure 6 **)** were detected, and the overall number of adipocytes decreased for P20–P40 passages. As expected, vascular structures were altered after a mechanical dissociation.

### Immunomodulatory properties on monocytes and macrophages

**FIGURE 7:**
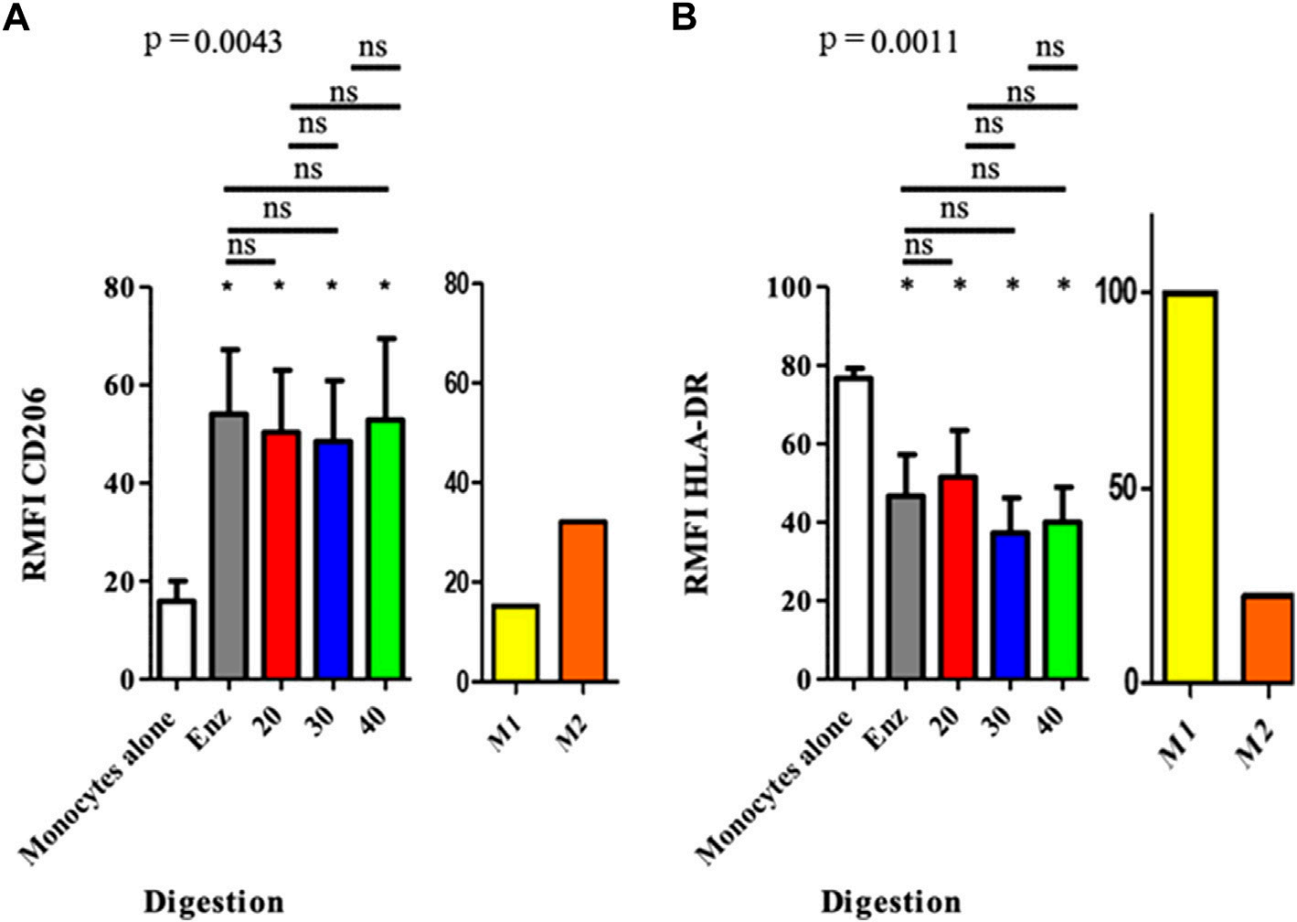
Polarization of macrophages after co-culture with ASCs at the end of P0. The results are expressed as the fluorescence ratio of the TEST tube/FMO tube, obtained by mono parametric test. If the ratio is < 1, the marker is not expressed; between 1 and 2, the expression of the marker cannot be concluded, and 2 the marker is expressed. *p < 0.05, **p < 0.001, and ***p < 0.0001; ns: not significant. ASCs: adipose-derived mesenchymal stem cells.

Macrophages co-cultured with ASCs from all the different SVFs significantly increased their expression of the CD206 marker, compared to the control group. The RMFI values were the following: for the control group 15.87 (± 10.99); for the enzymatically isolated SVF 54.06 (± 34.70, *p* = 0.0156); and for the mechanically isolated SVF group 50.32 (± 33.4, *p* = 0.0156), 48.47 (± 32.80, *p* = 0.0156), and 52.87 (± 43.80, *p* = 0.0156) for P20, P30, and P40, respectively. No difference was observed between the different co-culture conditions. However, the expression of the HLA-DR marker was significantly decreased for all conditions, compared to that of the monocyte control group. We found an RMFI of 76.66 (± 6.71) for monocytes alone; 46.69 ( ± 27.91, *p* = 0.0343); and in the enzymatically isolated SVF group; 51.48( ± 31.65, *p* = 0.0343), 37.25 ( ± 23.69, *p* = 0.0223), and 40.02 ( ± 23.50; *p* = 0.0223) for P20, P30, and P40 mechanically isolated SVF subgroups, respectively. However, like for CD206 and CD163, there was no difference between the ASCs from the different methods of SVF isolation. The expression level increased for CD206 and decreased for HLA-DR, and co-cultured ASCs and monocytes tend to lead to an M2 phenotype. There was no difference between ASCs of different mechanically isolated SVF and enzymatically isolated SVF samples, regarding the polarization level of monocytes/macrophages (Figure 7). This means that a greater number of intersyringe passages increased the ECM destruction, as demonstrated by a progressive diminution of residues in the filter (Supplementary material S1), and did not release more cells as we thought it would but instead proportionally destroyed more of it.

## Discussion

The use of adipose tissue in surgery is both an ancient practice and a growing concept. Initially used as a filler substance, the discovery of ASCs by Zuk et al. (2002) and then the development of knowledge of this tissue, the cells that make it up and their properties have multiplied the medical indications. Seeking to concentrate the cells and purify the substrate for reinjection, several techniques have followed one another to optimize the grafting of adipose tissue. The Coleman technique is the most commonly used in current practice, allowing clean adipose tissue to be reinjected, and washed from this waste, to optimize engraftment. With a view to performing tissue regeneration, the objective is to overcome the filler effect of TA to concentrate the cells of interest (ASC, macrophages, and endothelial cells). Other techniques such as Microfragmented adipose tissue or LIPOGEMS^®^ can reduce sub-millimeter adipocyte clusters (Bianchi et al., 2013). They have been shown to produce a reinjection substrate containing ASCs (Maioli et al., 2014) endothelial cells, pericytes, and anti-inflammatory and anti-angiogenic properties (Ceserani et al., 2016; Bouglé et al., 2018; Ragni et al., 2022). The indications are very promising for intra-tissue reinjection (Tremolada et al., 2016; Naldini et al., 2018; Ceresa et al., 2022), especially intra-articular injection (Cattaneo et al., 2018; Filardo et al., 2022), where the reinjected product combines the functionality of the cells of interest with a mechanical effect. However, from the perspective of tissue engineering, an SVF seems even more interesting because it further reduces the clusters and the filler effect of adipocytes by concentrating the cells of interest. An SVF obtained with the least possible manipulation, directly in the operating room and at a lower cost is of capital interest in current clinical practice and opens the door to new perspectives in the laboratory because of the number of cells obtained and their functionality.

We optimized the NG technique and demonstrated that the increase of intersyringe passages reduced the SVF final number of cells, adjusted per gram of AT, along with a reduction of ASC phenotypes and clonogenic cells (CFU-F). Interestingly, compared to enzymatically isolated ones, there were more cells in mechanically isolated SVFs. This was quite unexpected, compared to previous findings (Shah et al., 2013; Markarian et al., 2014; Chaput et al., 2016). It could be explained by the fact that, in order to perform flow cytometry on SVF, we had to use a 100-μm strainer to avoid clogging the cytometer. Second, enzymatic digestion had been described by Van Dongen et al., 2016 and by Mashiko et al. (2017) with only one collagenase. The cocktail was made of type IV collagenase, dispase, and DNase to avoid cellular aggregates and magnesium chloride, which acted as an enzymatic activator. This cocktail enabled us to analyze more thoroughly the composition of each SVF. Other studies compared the enzymatic activator to mechanical dissociation after straining for cytometric analyses (Tonnard et al., 2013; Aronowitz et al., 2015; Chaput et al., 2016; Mashiko et al., 2017), without taking into account this inherent bias. It should be noted that, unlike its original description, intersyringe emulsification was not filtered through a 500-μm sterile nylon: indeed, the pore size (500 μm) is too large to retain different cell populations of AT and would only retain fibrous and vascular structures not eliminated after digestion, as we previously demonstrated (Bertheuil et al., 2016). The number of cells per gram of initial AT (cpg), obtained after enzymatic digestion in our study (208 575 cpg ± 318774), was lower than Chaput’s (600 000 cpg) (Chaput et al., 2016) and Yoshimura’s (1 310 000 cpg) (Yoshimura et al., 2006). However, even with the same collagenase, the number of cells remains higher than that of Aronowitz and Ellenhorn, 2013 (101 061 cpg) who used the Cytori device. Chaput obtained fewer cells than we did (40 000 vs. 291,272 cpg) (Chaput et al., 2016) as Tonnard (19750 cpg) (Tonnard et al., 2013) without enzymatic digestion of mechanically isolated SVF. In contrast, Mashiko found more cells (around 600 000 cpg) with additional enzymatic digestion (Mashiko et al., 2017). This demonstrates the necessity of an additional enzymatic digestion step, in order to get a better representation of the isolated cell population and amount. Regarding the number of ASCs per gram of initial AT, our results found 55 643 ± 54 049 cpg with the M30 technique. In order to compare these results with the literature, we used the same gating strategy as Chaput (Chaput et al., 2016) and Mashiko (Mashiko et al., 2017). From their work, we can consider CD45neg CD31neg CD34pos cells as ASCs. Preliminary unpublished results of our laboratory (Bertheuil, 2017) confirmed these CD45neg CD31neg CD34pos also express CD73, CD90, and CD105 specific markers of MSCs. **(** Supplementary material S2). Mashiko et al. (2017) found more cells (150 000 cpg) but used a smaller syringe of 2.5 vs. 10 ml, which could have had an impact on the pressure put on the cells, when they are pushed through the connector. Through second enzymatic digestion, we found 57.58% ± 11.38 of ASCs in SVF which is more than what is described in the literature: 21.45% ± 2.52 (Chaput et al., 2016); 10.68% (Aronowitz and Ellenhorn, 2013), same enzyme but using the Celution device for digestion; 14% (Güven et al., 2012), with Sepax device. With mechanically isolated SVF obtained with P30, we found 30,52% ± 13.09 ASCs, which is in line with Mashiko’s findings of near 35% (Mashiko et al., 2017) and with Chaput’s findings of 38.11% ± 5.14 (Chaput et al., 2016) using a similar ASC gating strategy. It is interesting to note that Tonnard only found 5% (Tonnard et al., 2013) with ASC staining with only CD34^+^. We found an 8.02% ± 6.11 rate of endothelial cells with our enzymatic digestion, comparable to that of Chaput (7.64% ± 1.50) (Chaput et al., 2016). Although, unlike us, collagenase was used alone in that study, which means that enzymatic digestion had few impacts upon the vascular network. Sundarraj’s rate of 3% ± 1 is in line with our findings (SundarRaj et al., 2015). On the other hand, with P30 passages, we found 51.09% ± 16.42 of endothelial cells, which is much higher than that of Guven (25%) (Güven et al., 2012), Mashiko (25%–30%) (Mashiko et al., 2017), or Chaput (Chaput et al., 2016) studies. Interestingly, regarding CFU-F, enzymatically isolated SVF had a lower ASC rate (22.11% ± 7.44) than P20 and P30 mechanically isolated SVF. These results are the opposite of Chaput’s results of 36.74% ± 4.96 after enzymatic digestion and 17.79% ± 4.29 after mechanical dissociation, possibly due to the absence of second enzymatic digestion and an underestimation of mechanically isolated SVF content (Chaput et al., 2016). Aronowitz et al. (2015)found only 16% of CFU-F with the Celution device (Aronowitz et al., 2015). In our study, with regards to the overall number of cells per gram of initial tissue and the number of ASCs, the P20 technique was superior to the P30 and P40 mechanically isolated SVF. It means that a greater number of passages between syringes increases the extracellular matrix disruption, as observed with fewer residues in the filter and not releasing as many cells as expected, but instead destroying these cells. In his work, Tonnard et al. (2013) claimed that there were no adipocytes in the reinjected product because of the absence of viable adipocytes, as assessed by direct fluorescence microscopy, after AM-calcein and DAPI staining. Here, we demonstrated the presence of adipocytes after mechanical dissociation, regardless of the number of passages between the syringes. This major difference is probably related to the confocal microscopy technique which, unlike fluorescence microscopy, is more precise and allows the visualization of a cell. Immunomodulatory properties of MSCs are well known but those of ASCs are less well documented, especially when obtained by mechanical dissociation (Mallis et al., 2021). A previous study (Chaput et al., 2016) showed that mechanically isolated SVF isolated ASCs presented a similar inhibition of proliferation of activated T-lymphocytes after one culture passage, compared to ASCs from enzymatically isolated SVF. We also assessed if ASCs from mechanically isolated SVF and enzymatically isolated SVF had similar immunomodulatory properties on macrophages, key players in tissue regeneration processes. When SVF is directly reinjected in a tissue, the immunomodulatory effect of ASCs on macrophages seems more relevant for regenerative purposes**.** We determined the effect of each ASC group based on the phenotype modifications of cocultured monocyte cells. The expression of three markers (CD206, CD163, and HLA-DR) was used to determine the polarization state of cells, allowing us to conclude that macrophages from mechanically isolated SVF had the same M2 orientated profile as those from enzymatic isolated SVF. M2-orientated polarization documented here is an additional element to comfort the application of the NG technique, with mechanically isolated SVF to seed engineered tissues and promote *in vivo* regeneration. Regarding immunomodulatory properties of ASCs, depending on the type of isolation methodology, a previous study showed that the mechanically isolated ASCs demonstrated a similar inhibition of proliferation of activated T lymphocytes in CFSE, after one culture passage (Chaput et al., 2016), as it is known for MSCs (Mallis et al., 2021). This is particularly interesting for cell therapy purposes. However, when directly reinjecting SVF in a tissue, meaning without any laboratory step between clinical procurement and mechanical processing, the immunomodulatory effect of ASCs on macrophages seems more relevant. In this work, it was difficult to get an exhaustive profile demonstrating a pro-inflammatory (M1) or anti-inflammatory (M2) environment, especially *in vivo* where a control group is difficult to achieve. This determination is based on the cell’s phenotype, its phagocytosis ability, and its secretory profiles. We used a shortcut commonly described in studies that analyzes only surface markers of macrophages. The variation in the intensity of expression of three markers (CD206, CD163, and HLA-DR) was used to determine the polarization state of cells. We wanted to look at the macrophage’s status within the reinjected SVF and the immunomodulatory effect of ASCs after one passage in culture on macrophages *in vitro*. First of all, the *in vivo* marking (CD206, CD163, and HLA-DR) on myeloid sub-populations allowed us to conclude that macrophages from mechanically isolated SVF had a more anti-inflammatory profile than those from enzymatic isolated SVF. Nevertheless, we could not conclude their polarization without measuring the specific cytokines as explained previously. This is in line with the literature where it is now clearly accepted that MSCs promote a macrophage M2 profile. Dong et al. (2013) showed that CD206 and CD163 increases in PCR, when adipose tissue is cultured with SVF in mice, tend to show that ASCs give macrophages an M2 profile. Moreover, Kim and Hematti (2009) also demonstrated that co-cultured macrophages and ASCs have a higher expression of CD206 and an increased secretion of IL-10 and IL-6, as long as reduced secretion of TNF alpha and IL-12. Adutler-Lieber et al. (2013) showed that in a co-culture of monocytes and ASCs, there is an increased expression of CD-206 and CD-163, increased secretion of IL-13, IL-4, VEGF, and IL-10, and reduced secretion of IL-1, IL-12, TNF alpha, IL-17, and INF gamma. Rybalko et al. (2017) showed a proportional increase of CD-206 within the ASC coculture when the macrophage/ASC ratio reached 5:1. Our findings are not statistically significant even though we used a 4/1 ratio in culture. However, immunomodulatory properties of ASCs obtained by mechanical dissociation are not known. We, therefore, preferred to initiate a co-culture with non-oriented monocytes in case these types of ASCs orient macrophages toward an M1 profile. The demonstration of their capacity to orient macrophages toward an M2 profile would have been strengthened if the same results had been demonstrated on macrophages pre-oriented toward an M1 profile instead of neutral monocytes. Nevertheless, the M2 polarization of ASCs obtained by mechanical dissociation seems interesting for regenerating tissues. Moreover, *in vivo*, there are no M1 or M2 macrophages which are laboratory creations but a continuum of macrophages going from M1 to M2.

## Conclusion

The SVF use represents a major strategy in the clinical ECM recellularization approach: a large source of autologous progenitor cells, both in cell types and number, being easily harvested with the minimal morbidity of liposuction or lipectomies—classically used in plastic and aesthetic surgery—as well as an easy and cost-efficient extraction. The so-obtained SVF can then be processed to cellularize engineered constructs, either directly in an operating theater, or to be used in a bioreactor. The great advantage is also to avoid all the regulatory constraints as cells are autologous and with very limited manipulation. Our work is the first to precisely detail this human adipose-derived SVF composition and optimize the mechanical technique, compared to the enzymatic approach. It gives an exhaustive listing of the different cellular actors involved. However, interaction mechanisms between the different cell sub-populations and host tissues are still to define. This work suggests modifying the existing NG technique with 20 intersyringe passages is optimal. For regenerative purposes in the operating room, mechanical SVF will play an important role in the therapeutic arsenal of the regenerative surgeon because of the M2 macrophages’ anti-inflammatory and pro-angiogenic abilities, which are true architects of regeneration. It is undeniable that there is a clinical benefit when mechanical SVF contains myeloid cells with an anti-inflammatory profile and the same overall number of cells as enzymatic SVF. Moreover, mechanically isolated SVF has a greater number of endothelial cells, which promotes a pro-angiogenic environment and a greater number of immunomodulatory/anti-inflammatory ASCs, with greater clonogenicity. Given its low cost of production, especially compared to enzymatic digestion in a laboratory or use of the Celution device (2400$ for one dissociation) (Aronowitz and Ellenhorn, 2013), mechanical SVF after 20 passages between two syringes appears as a very efficient and affordable technique. Furthermore, their clonogenicity ability is not altered after P0 culture which means mechanically isolated SVF could also become a reference to produce ASCs for cell therapy.

## Data Availability

The raw data supporting the conclusion of this article will be made available by the authors, without undue reservation.

## References

[B1] Adutler-Lieber S. Ben-Mordechai T. Naftali-Shani N. Asher E. Loberman D. Raanani E. (2013). Human macrophage regulation via interaction with cardiac adipose tissue-derived mesenchymal stromal cells. J. Cardiovasc. Pharmacol. Ther. 18, 78–86. 10.1177/1074248412453875 22894882

[B2] Aronowitz J. A. Ellenhorn J. D. I. (2013). Adipose stromal vascular fraction isolation: A head-to-head comparison of four commercial cell separation systems. Plastic Reconstr. Surg. 132, 932e–9e. 10.1097/PRS.0b013e3182a80652 24281640

[B3] Aronowitz J. A. Lockhart R. A. Hakakian C. S. (2015). Mechanical versus enzymatic isolation of stromal vascular fraction cells from adipose tissue. SpringerPlus 4, 713. 10.1186/s40064-015-1509-2 26636001 PMC4656256

[B4] Badylak S. F. (2002). The extracellular matrix as a scaffold for tissue reconstruction. Semin. Cell Dev. Biol. 13, 377–383. 10.1016/s1084952102000940 12324220

[B5] Badylak S. (2016). Perspective: Work with, not against, biology. Nature 540, S55. 10.1038/540S55a 27926701

[B6] Bertheuil N. Chaput B. Berger-Müller S. Ménard C. Mourcin F. Watier E. (2016). Liposuction preserves the morphological integrity of the microvascular network: Flow cytometry and confocal microscopy evidence in a controlled study. Aesthet. Surg. J. 36, 609–618. 10.1093/asj/sjv209 26530477

[B7] Bertheuil N. Chaput B. Ménard C. Varin A. Garrido I. Grolleau J. L. (2015). Adipose-derived stromal cells: History, isolation, immunomodulatory properties and clinical perspectives. Ann. Chir. Plast. Esthet. 60, 94–102. 10.1016/j.anplas.2014.09.014 25446469

[B8] Bertheuil N. Chaput B. Ménard C. Varin A. Laloze J. Watier E. (2019). Adipose mesenchymal stromal cells: Definition, immunomodulatory properties, mechanical isolation and interest for plastic surgery. Ann. Chir. Plast. Esthet. 64, 1–10. 10.1016/j.anplas.2018.07.005 30126741

[B9] Bertheuil N. (2017). Le tissu adipeux : Approfondissement des connaissances fondamentales du tissu et de son compartiment vasculaire stromal, intérêt clinique pour la chirurgie plastique. Available at: https://www.theses.fr/2017REN1B051 (Accessed July 9, 2022).

[B10] Bianchi F. Maioli M. Leonardi E. Olivi E. Pasquinelli G. Valente S. (2013). A new nonenzymatic method and device to obtain a fat tissue derivative highly enriched in pericyte-like elements by mild mechanical forces from human lipoaspirates. Cell Transpl. 22, 2063–2077. 10.3727/096368912X657855 23051701

[B11] Bouglé A. Rocheteau P. Hivelin M. Haroche A. Briand D. Tremolada C. (2018). Micro-fragmented fat injection reduces sepsis-induced acute inflammatory response in a mouse model. Br. J. Anaesth. 121, 1249–1259. 10.1016/j.bja.2018.03.032 30442252

[B12] Cattaneo G. De Caro A. Napoli F. Chiapale D. Trada P. Camera A. (2018). Micro-fragmented adipose tissue injection associated with arthroscopic procedures in patients with symptomatic knee osteoarthritis. BMC Musculoskelet. Disord. 19, 176. 10.1186/s12891-018-2105-8 29848328 PMC5977739

[B13] Ceresa C. Borrone A. Fracchia L. Rinaldi M. Marchetti A. Tremolada C. (2022). Lipoaspirate shows *in vitro* potential for wound healing. Pharmaceutics 14, 447. 10.3390/pharmaceutics14020447 35214179 PMC8878490

[B14] Ceserani V. Ferri A. Berenzi A. Benetti A. Ciusani E. Pascucci L. (2016). Angiogenic and anti-inflammatory properties of micro-fragmented fat tissue and its derived mesenchymal stromal cells. Vasc. Cell 8, 3. 10.1186/s13221-016-0037-3 27547374 PMC4991117

[B15] Chaput B. Bertheuil N. Escubes M. Grolleau J.-L. Garrido I. Laloze J. (2016). Mechanically isolated stromal vascular fraction provides a valid and useful collagenase-free alternative technique: A comparative study. Plastic Reconstr. Surg. 138, 807–819. 10.1097/PRS.0000000000002494 27307342

[B16] Crapo P. M. Gilbert T. W. Badylak S. F. (2011). An overview of tissue and whole organ decellularization processes. Biomaterials 32, 3233–3243. 10.1016/j.biomaterials.2011.01.057 21296410 PMC3084613

[B17] Dominici M. Le Blanc K. Mueller I. Slaper-Cortenbach I. Marini F. Krause D. (2006). Minimal criteria for defining multipotent mesenchymal stromal cells. The International Society for Cellular Therapy position statement. Cytotherapy 8, 315–317. 10.1080/14653240600855905 16923606

[B18] Dong Z. Peng Z. Chang Q. Lu F. (2013). The survival condition and immunoregulatory function of adipose stromal vascular fraction (SVF) in the early stage of nonvascularized adipose transplantation. PLoS ONE 8, e80364. 10.1371/journal.pone.0080364 24260375 PMC3832367

[B19] Duisit J. Amiel H. Wüthrich T. Taddeo A. Dedriche A. Destoop V. (2018). Perfusion-decellularization of human ear grafts enables ECM-based scaffolds for auricular vascularized composite tissue engineering. Acta Biomater. 73, 339–354. 10.1016/j.actbio.2018.04.009 29654989

[B20] Filardo G. Tschon M. Perdisa F. Brogini S. Cavallo C. Desando G. (2022). Micro-fragmentation is a valid alternative to cell expansion and enzymatic digestion of adipose tissue for the treatment of knee osteoarthritis: A comparative preclinical study. Knee Surg. Sports Traumatol. Arthrosc. 30, 773–781. 10.1007/s00167-020-06373-y 33464397

[B21] Gentile P. Sterodimas A. Pizzicannella J. Dionisi L. De Fazio D. Calabrese C. (2020). Systematic review: Allogenic use of stromal vascular fraction (SVF) and decellularized extracellular matrices (ECM) as advanced therapy medicinal products (ATMP) in tissue regeneration. Int. J. Mol. Sci. 21, E4982. 10.3390/ijms21144982 32679697 PMC7404290

[B22] Güven S. Karagianni M. Schwalbe M. Schreiner S. Farhadi J. Bula S. (2012). Validation of an automated procedure to isolate human adipose tissue-derived cells by using the Sepax® technology. Tissue Eng. Part C. Methods 18, 575–582. 10.1089/ten.TEC.2011.0617 22372873 PMC3401386

[B23] Kim J. Hematti P. (2009). Mesenchymal stem cell-educated macrophages: A novel type of alternatively activated macrophages. Exp. Hematol. 37, 1445–1453. 10.1016/j.exphem.2009.09.004 19772890 PMC2783735

[B24] Lo Furno D. Tamburino S. Mannino G. Gili E. Lombardo G. Tarico M. S. (2017). Nanofat 2.0: Experimental evidence for a fat grafting rich in mesenchymal stem cells. Physiol. Res. 66, 663–671. 10.33549/physiolres.933451 28406706

[B25] Maioli M. Rinaldi S. Santaniello S. Castagna A. Pigliaru G. Delitala A. (2014). Radioelectric asymmetric conveyed fields and human adipose-derived stem cells obtained with a nonenzymatic method and device: A novel approach to multipotency. Cell Transpl. 23, 1489–1500. 10.3727/096368913X672037 24044359

[B26] Mallis P. Michalopoulos E. Chatzistamatiou T. Giokas C. S. (2021). Interplay between mesenchymal stromal cells and immune system: Clinical applications in immune-related diseases. Explor. Immunol. 1, 112–139. 10.37349/ei.2021.00010

[B27] Markarian C. F. Frey G. Z. Silveira M. D. Chem E. M. Milani A. R. Ely P. B. (2014). Isolation of adipose-derived stem cells: A comparison among different methods. Biotechnol. Lett. 36, 693–702. 10.1007/s10529-013-1425-x 24322777

[B28] Mashiko T. Wu S.-H. Feng J. Kanayama K. Kinoshita K. Sunaga A. (2017). Mechanical micronization of lipoaspirates: Squeeze and emulsification techniques. Plastic Reconstr. Surg. 139, 79–90. 10.1097/PRS.0000000000002920 27627056

[B29] Naldini G. Sturiale A. Fabiani B. Giani I. Menconi C. (2018). Micro-fragmented adipose tissue injection for the treatment of complex anal fistula: A pilot study accessing safety and feasibility. Tech. Coloproctol. 22, 107–113. 10.1007/s10151-018-1755-8 29453515

[B30] Ragni E. Viganò M. Torretta E. Perucca Orfei C. Colombini A. Tremolada C. (2022). Characterization of microfragmented adipose tissue architecture, mesenchymal stromal cell content and release of paracrine mediators. J. Clin. Med. 11, 2231. 10.3390/jcm11082231 35456324 PMC9026471

[B31] Raposio E. Caruana G. Bonomini S. Libondi G. (2014). A novel and effective strategy for the isolation of adipose-derived stem cells: Minimally manipulated adipose-derived stem cells for more rapid and safe stem cell therapy. Plastic Reconstr. Surg. 133, 1406–1409. 10.1097/PRS.0000000000000170 24867723

[B32] Raposio E. Caruana G. Petrella M. Bonomini S. Grieco M. P. (2016). A standardized method of isolating adipose-derived stem cells for clinical applications. Ann. Plast. Surg. 76, 124–126. 10.1097/SAP.0000000000000609 26418805

[B33] Rybalko V. Hsieh P.-L. Ricles L. M. Chung E. Farrar R. P. Suggs L. J. (2017). Therapeutic potential of adipose-derived stem cells and macrophages for ischemic skeletal muscle repair. Regen. Med. 12, 153–167. 10.2217/rme-2016-0094 28244825 PMC5348723

[B34] Scarritt M. E. Pashos N. C. Bunnell B. A. (2015). A review of cellularization strategies for tissue engineering of whole organs. Front. Bioeng. Biotechnol. 3, 43. 10.3389/fbioe.2015.00043 25870857 PMC4378188

[B35] Shah F. S. Wu X. Dietrich M. Rood J. Gimble J. M. (2013). A non-enzymatic method for isolating human adipose tissue-derived stromal stem cells. Cytotherapy 15, 979–985. 10.1016/j.jcyt.2013.04.001 23725689

[B36] SundarRaj S. Deshmukh A. Priya N. Krishnan V. S. Cherat M. Majumdar A. S. (2015). Development of a system and method for automated isolation of stromal vascular fraction from adipose tissue lipoaspirate. Stem Cells Int. 2015, 1–11. 10.1155/2015/109353 PMC4475713 26167182

[B37] Tan Y. Landford W. N. Garza M. Suarez A. Zhou Z. Coon D. (2019). Complete human penile scaffold for composite tissue engineering: Organ decellularization and characterization. Sci. Rep. 9, 16368. 10.1038/s41598-019-51794-6 31704952 PMC6841966

[B38] Tonnard P. Verpaele A. Peeters G. Hamdi M. Cornelissen M. Declercq H. (2013). Nanofat grafting: Basic research and clinical applications. Plastic Reconstr. Surg. 132, 1017–1026. 10.1097/PRS.0b013e31829fe1b0 23783059

[B39] Tremolada C. Colombo V. Ventura C. (2016). Adipose tissue and mesenchymal stem cells: State of the art and Lipogems® technology development. Curr. Stem Cell Rep. 2, 304–312. 10.1007/s40778-016-0053-5 27547712 PMC4972861

[B40] van Dongen J. A. Stevens H. P. Parvizi M. van der Lei B. Harmsen M. C. (2016). The fractionation of adipose tissue procedure to obtain stromal vascular fractions for regenerative purposes. Wound Repair Regen. 24, 994–1003. 10.1111/wrr.12482 27717133

[B41] Wang L. Johnson J. A. Zhang Q. Beahm E. K. (2013). Combining decellularized human adipose tissue extracellular matrix and adipose-derived stem cells for adipose tissue engineering. Acta Biomater. 9, 8921–8931. 10.1016/j.actbio.2013.06.035 23816649 PMC3965366

[B43] Yoshimura K. Shigeura T. Matsumoto D. Sato T. Takaki Y. Aiba-Kojima E. (2006). Characterization of freshly isolated and cultured cells derived from the fatty and fluid portions of liposuction aspirates. J. Cell. Physiol. 208, 64–76. 10.1002/jcp.20636 16557516

[B42] Zuk P. A. Zhu M. Ashjian P. De Ugarte D. A. Huang J. I. Mizuno H. (2002). Human adipose tissue is a source of multipotent stem cells. Mol. Biol. Cell 13, 4279–4295. 10.1091/mbc.e02-02-0105 12475952 PMC138633

