# Supplementary figures and images for: Modified nanofat grafting: Stromal vascular fraction simple and efficient mechanical isolation technique and perspectives in clinical recellularization applications
Source: Front Bioeng Biotechnol. 2022 Sep 13;10:895735. doi: 10.3389/fbioe.2022.895735 (PMC9513316; doi:10.3389/fbioe.2022.895735)

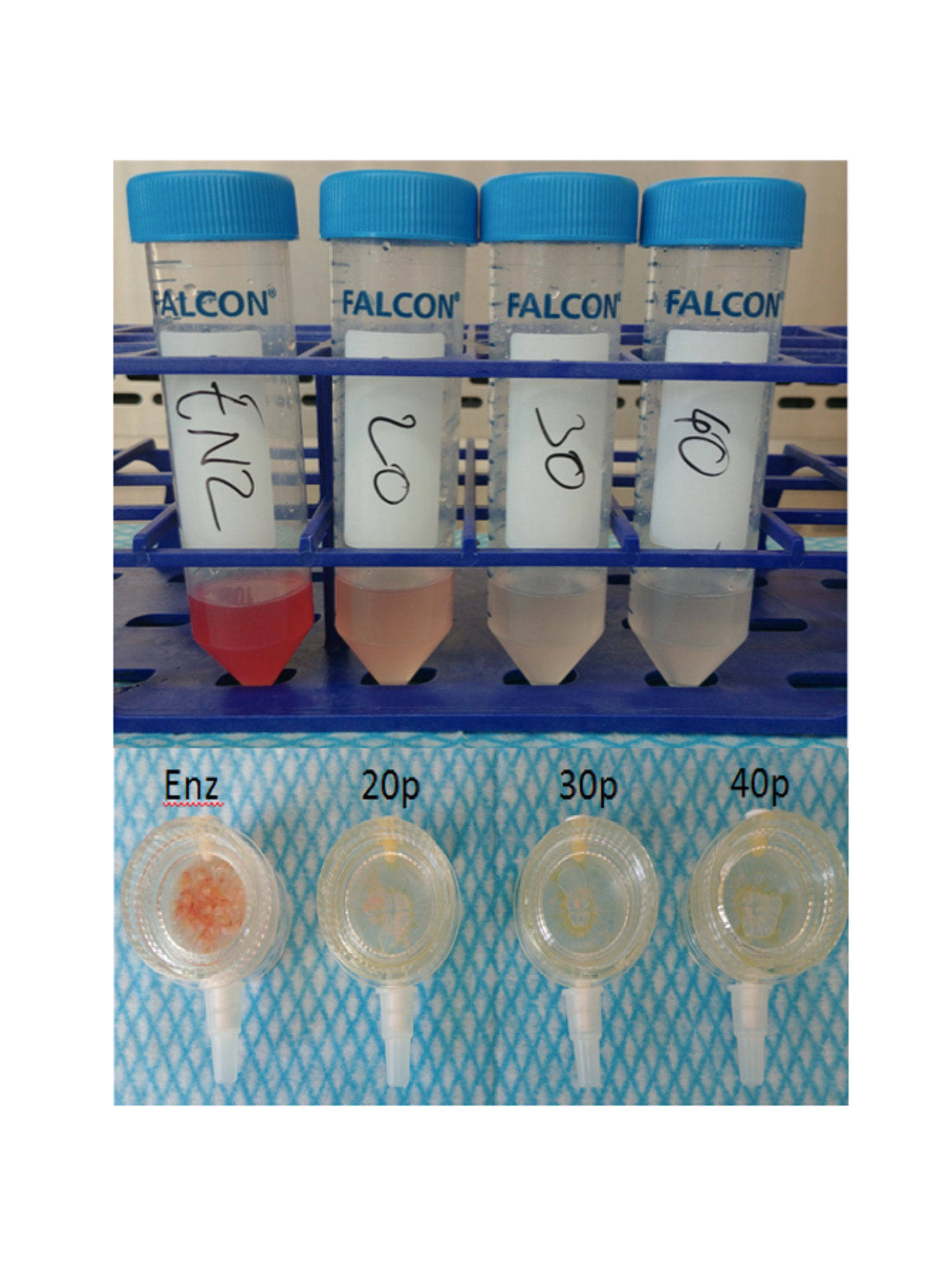

Supplement: Supplementary file 1 [file Image1.TIFF]

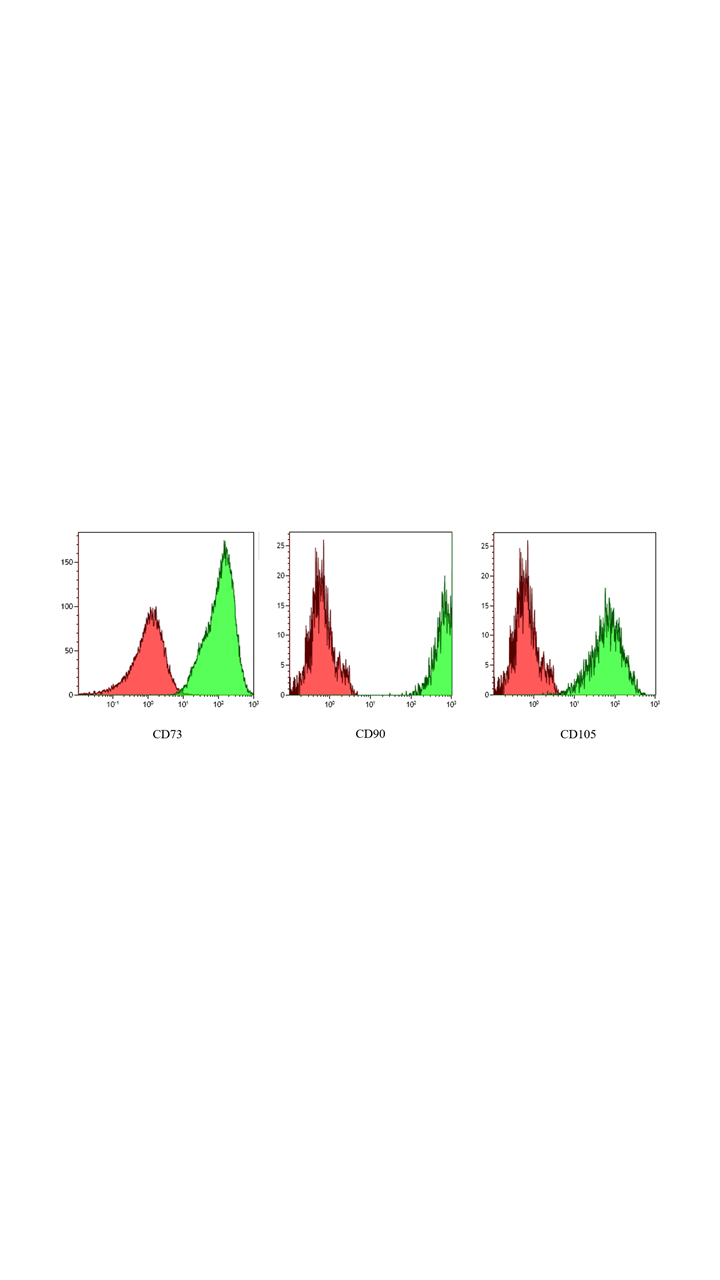

Supplement: Supplementary file 2 [file Image2.TIF]
